# Twelve months into a feasibility trial: reflections on three experiences of public and patient involvement in research

**DOI:** 10.12688/hrbopenres.13205.1

**Published:** 2021-01-28

**Authors:** Robert Joyce, Christopher P. Dwyer, Sinéad M. Hynes

**Affiliations:** 1 School of Health Sciences, National University of Ireland, Galway, Galway, Ireland

**Keywords:** public and patient involvement, PPI, involvement, multiple sclerosis

## Abstract

In this Open Letter we present reflections from three different perspectives on the integration of public and patient involvement (PPI) in a research trial. We reflect on the experience of having a patient employed as a contract researcher, with no prior research experience, on a feasibility trial of cognitive rehabilitation in multiple sclerosis. This Open Letter is written by the PPI research team member with reflections from a researcher on the trial and the principle investigator. We will discuss some of the changes made and the impacts that have been resulted from of PPI input into the trial. We focus on PPI involvement in participant recruitment, the development of trial material, integration of PPI along the research cycle, and collaboration. We hope that this Open Letter will encourage principle investigators and groups to include PPI members as part of the research team and help patients and members of the public understand what the experience of PPI members is like.

## Disclaimer

The views expressed in this article are those of the authors. Publication in HRB Open Research does not imply endorsement by the Health Research Board of Ireland.

## Introduction

Patient and public involvement (PPI) can be considered a relatively new method of enhancing health-related research programs. Though research on the practice has highlighted the benefits of this integration over the past five to ten years, the literature is lacking with respect to the personalised experience(s) of PPI member involvement. The following reflections present personalised accounts of a PPI member’s experience of working with a research team during their development of a randomised controlled trial for a cognitive occupation-based program for people with multiple sclerosis (COB-MS). We present three different perspectives: the patient, the post-doctoral researcher, and the principle investigator.

## Patient perspective

Every fortnight, our research team (principal investigator [PI], the post-doc and the research assistants) meet to discuss progress on the COB-MS Trial. This meeting keeps everyone updated and also allocates tasks for the upcoming weeks and months. My role at these meeting is to be the voice of the patient.

At these meetings there are many examples of how I share my lived experience of multiple sclerosis (MS). In this letter I chose two examples. The first highlighting the different knowledge areas of the team members, and the second demonstrating access into the patient community.

### Knowledge scope

At one of these meetings, the PI said they had prepared the Patient Participant Manual for the study and they had a sample of the booklet. It was beautiful. For me it looked like something I would be proud to have in my bookcase or even on the coffee table. The production values were excellent. As a person with MS for 28 years, however, looking at this as a tool for making the intervention more effective for the participants, I knew it was only fit for the bin. Of course, I shared my opinion with the team - highlighting that it would not lie flat on the table, the pages were too slippery, and the gloss made it difficult to see what was written on the pages.

It surprised the team. At no stage did they realise the document they had produced would not be suitable for people like me, and why should they? No one else had my lived experience. Even though they knew all about MS, its symptoms and prognosis, they never lifted a book with MS hands.

Once they recovered from the surprise, we got down to the secondary benefit of having the patient at the table - I could share what I needed to make the manual more suitable. Some of the things I suggested were the importance of the type of paper, the binding, location of the page numbers, font and spacing – plus extras like a pouch for loose pages and extra forms (just in case). We then shared this format, along with the original, with the Patient Advisory Panel. They agreed with my opinion and added a few more suggestions.

What a success and a perfect example of why research should have a patient or potential recipient of a new therapy involved at all stages of the research. The researcher doesn’t know what they don’t know; and it is only through having the input of someone with the lived experience at the table they will know if they are going down the wrong path.

### Access to the community

At another meeting, we were discussing the recruitment of participants to the trial. This is always difficult, from what I am told, and when we had a small response to our initial press release, we began to talk about how else we can get in front of more people with MS.

Leaflets and posters in MS Society and GP offices was a start. Utilising my connection with a local radio station (Connemara FM), I did an interview about the study, my role in it and the type of people we needed. This brought in a few more people and revealed the power of the ‘patient story’. Subsequently, I used my connections within the MS community to get interviews with local and national press (e.g. Irish Independent, Irish Examiner and Farmers Journal, to name a few), as well as radio in areas we needed more participants.

The impact on recruitment was immediate. Within several weeks, we had enough to start the next stage of the trial and prepared to start the therapy. *Why was this successful?* I believe when you hear or read of someone who is going through a similar experience to your own, you form a bond, a camaraderie, which strengthens the trust you have in the message they share. I am one of them; and if I believe in it, then, perhaps, it will work for them, too.

### Embedded patient collaboration

Imagine an architect only asking the user of the building when the structure is nearly complete, where to locate the light switches, meanwhile none of the doors are wide enough for the user’s wheelchair. The recipient of the product, service, or therapy must be part of the process.

Developing anything new, which should have a long and useful life needs to be a collaborative venture – with all the stakeholders at the table, sharing equal status. We need more than engagement or participation, as these are additions to research, and not essential parts of the process. We need to be collaborators; patients don’t want to be there just to tick the box – *we* want to be there from the start, taking part in every decision.

The COB-MS program is about to be delivered to participants and the new manual will be used, which we hope will show people receiving the intervention that the research team understands what it is like to have MS and *our* particular needs.

I have shared two examples in this letter of how patient involvement has altered the course of this study. Some were small changes – and others bigger; but each one demonstrates to the participants in the trial that we understand what it is like to have MS. Every action is looked at through the lens of someone with the illness, who knows the role it plays in their daily experience. The challenge is how to measure the impact of each one of these on the trial.

In the last few months, this trial has also had to face the complication of the COVID-19 pandemic and its impact on research. I am an active part of the MS community here in Ireland, as well as in the UK and USA. I knew since its arrival the impact it would have on our study. Delivering the intervention in person would be an immense challenge. Online delivery would be the only route for most people with MS, because of the increased risk for this patient cohort.

Based on this knowledge, the team started to prepare for the possibility of changing the delivery method. We met with the PPI Advisory Panel to get their opinion and they agreed with the change. Moreover, the panel shared its experience of online therapies and groups, which helped us design the new format. This was combined with recommendations made in past, relevant research regarding how to use online platforms like that we are now using to deliver the program.

Once again, because a patient is ‘at the table’, it is possible to pre-empt potential challenges. *We* are also invested in the project, as we need it to be a success; and so, we can also provide concrete recommendations on how to proceed. We want and need to be collaborators. There for every decision, ensuring the focus remains on the patient first, with the goal of a better quality of life for those who receive the therapy.

## Post-doctoral researcher experience

I’ve been conducting research with human participants for about 15 years now and one thing I can say about it is that recruitment is always a tricky process – especially when recruiting a very specific cohort, such as people living with MS. Consistent with previous efforts to recruit for other interventions, the research team and I proposed to advertise the trial through relevant newsletters and websites for people living with MS (e.g. *MS Ireland*), through occupational therapists working with us to deliver the intervention, as well as posters and information leaflets posted in relevant clinics. Being cognisant of the modern world, we also made a point to advertise through social media. We also developed a press release for our trial through our university’s PR department, in the hope that either local or national press might pick our study up. Though we garnered fair interest through referrals from specific societies and services, unfortunately, we didn’t gain much interest in light of our press release. It seemed something was missing. 

As I believe now, that ‘something missing’ was, in fact, a story to tell. Perhaps, from a researcher’s standpoint, the most important thing to convey about participating in an intervention, like COB-MS, is a general idea about who the program is for, what is involved, how it might help and how to make contact. Though this method is straight to the point, truth be told, it probably lacks that ‘human’ touch in which people can more easily ‘connect’ with the message – much like a story. 

When our PPI member volunteered to take over the lead of advertising our study, the message ceased to be about the randomised nature of allocating participants, eligibility criteria or even about the outcome measures. All of that could be discussed later. For now, it was just about a single person with MS, telling their story – and when other people with MS heard that story (or even people who knew someone with MS – the message was shared), a connection was made and we started receiving emails and phone calls.

To be clear, our research team didn’t do anything wrong when we initially advertised the intervention. Indeed, the research team went about it much the same any other research team might. However, it’s not about what we did; rather, what we didn’t do sooner – and that’s giving the advertisement reins to our PPI from the very start. Sure, our PI or post-doc could have persevered to get on the radio or an interview for a paper; but that probably wouldn’t have done much to boost our sample either… because we don’t have MS. So, we don’t have that story to tell or the ability to connect with potential participants in that way; and that’s why PPI is so important to research programs like COB-MS. 

## Principal investigator experience

I believe that there are ethical reasons why patients should be included in the planning, design, and implementation of research. Within the scientific community, researchers tend to have strong opinions on the topic of PPI in research ( Boivin *et al.*, 2018). Though I have always valued PPI involvement in research, my “involvement” with PPI had been at somewhat of a distance before the COB-MS trial.

The effectiveness of PPI is strongest when people with lived experience of the condition being studied are involved as research partners ( Crocker *et al.*, 2018). When writing the funding application for the COB-MS feasibility trial, I wanted to be sure that there was a strong PPI through the trial. Though I anticipated that it might be a challenge to have a PPI member as a contract researcher, I believed that it would be key for this feasibility trial to have an experience-based expert contributing to decisions about the trial. It can be a challenge when expectations, values and assumptions of researchers and PPI members do not match and this can impact on the experience of both parties ( O’Shea *et al.*, 2019) but this can be addressed through discussion and compromise (most of the time!). 

PPI members as experts of their own condition, have experience that is invaluable in the design and running of clinical trials, as well as being aware of the needs, worries, and expectations of participants. Our PPI members are experience-based experts who contribute knowledge that is complementary to that of researchers on the trial ( Karazivan *et al.*, 2015) and have led to us making changes in a number of key areas. Two I will discuss here are 1) development of our participant handbook, and 2) participant recruitment. Having already read the reflections of the PPI contributor and the postdoc I will give my perspective (PI on the trial) of these examples.

### The participant handbook

The content of the handbook (which details the intervention) was developed prior to the beginning of the trial. Part of this process involved discussion. Feedback and focus groups with key stakeholders- in this case occupational therapists and people with MS. Once we updated and formatted the handbook, I was happy to get it printed off and ready for participants. A sample of the handbook was brought to the research team meeting for approval. Time had been put into the design and layout so I had not anticipated any issues with it. The researchers were happy with it, but it was our PPI contributor who immediately spotted a number of important issues that needed to be addressed before we printed the batch for our participants. Given the changes suggested, we decided to have a meeting with our wider PPI panel where we brought samples and discussed key issues that needed to be changed or included in the final version. These issues would not have been spotted were it not for the PPI member. The handbook is now being successfully used by participants in the trial and informal feedback has been very positive. 

### Participant recruitment

As we were recruiting participants, there were some geographical areas that we were finding it difficult to recruit from. We did a call through the MS Society and through the occupational therapists working in the area but were unable to recruit adequate numbers in some areas. Our PPI member decided to get involved in the recruitment process and took it upon themselves to do interviews for radio and print media, with a specific focus on the difficult to recruit areas. This input allowed people listening to hear the experience of someone living with the same condition and explain the research in a way that is meaningful and understandable to them. This effect on recruitment has been reported elsewhere with PPI interventions having a modest but significant increase the odds of participant enrolment ( Crocker *et al.*, 2018; Domecq *et al.*, 2014). There are still barriers that exist to recruiting under-served communities in research and clinical research has for the most part failed to adequately address this issue. Involving PPI members from these under-served communities could be a way of trying to increase the diversity of our participants ( Smits *et al.*., 2020). The INCLUDE roadmap, developed by the NIHR ( NIHR, 2020) provides an overview of the stages of the research process where researchers can act to include more diverse groups of people, particularly those defined as “under-served”. This is a goal that we have for future PPI work in MS. 

There are those who feel that in order to be taken seriously, we must evaluate involvement as we do other research processes, though this view is being debated more recently. If PPI is viewed as a part of the research process then maybe there is not the same need to measure its impact and we would be better served capturing the negative and positive aspects of the process instead ( Russell *et al.*, 2020). One clear reflection is that by having a strong PPI input we have never forgotten why we are doing the research we are doing, and how it might impact the lives of those we are serving through our research. 

## Data availability

No data are associated with this article.
