# Twelve months into a feasibility trial: reflections on three experiences of public and patient involvement in research

**DOI:** 10.12688/hrbopenres.13205.2

**Published:** 2021-07-22

**Authors:** Robert Joyce, Christopher P. Dwyer, Sinéad M. Hynes

**Affiliations:** 1 School of Health Sciences, National University of Ireland, Galway, Galway, Ireland

**Keywords:** public and patient involvement, PPI, involvement, multiple sclerosis

## Abstract

In this Open Letter we present reflections from three different perspectives on the integration of public and patient involvement (PPI) in a research trial. We reflect on the experience of having a patient employed as a contract researcher, with no prior research experience, on a feasibility trial of cognitive rehabilitation in multiple sclerosis. This Open Letter is written by the PPI research team member with reflections from a researcher on the trial and the principal investigator. We will discuss some of the changes made and the impacts that have been resulted from of PPI input into the trial. We focus on PPI involvement in participant recruitment, the development of trial material, integration of PPI along the research cycle, and collaboration. We hope that this Open Letter will encourage principal investigators and groups to include PPI members as part of the research team and help patients and members of the public understand what the experience of PPI members is like.

## Disclaimer

The views expressed in this article are those of the authors. Publication in HRB Open Research does not imply endorsement by the Health Research Board of Ireland.

## Introduction

Patient and public involvement (PPI) can be considered a relatively new method of enhancing health-related research programs. Though research on the practice has highlighted the benefits of this integration over the past five to ten years, the literature is lacking with respect to the personalised experience(s) of PPI member involvement. The following reflections present personalised accounts of a PPI member’s experience of working with a research team during their development of a randomised controlled trial for a cognitive occupation-based program for people with multiple sclerosis (COB-MS). We present three different perspectives: the patient, the post-doctoral researcher, and the principal investigator.

The patient is a common thread throughout the trial. Upon grant application, the MS Society of Ireland and the PPI Ignite project were advisors on how best to incorporate PPI. This led to the employment of an Assistant Researcher living with MS (from here on described as an Embedded Patient Researcher [EPR]), a Patient Advisory Panel and patients being members of the Trial Steering Committee and Trial Management Committee.

This letter focuses on how including a researcher with lived experience of the illness being researched has enhanced the quality of the trial. While non-patient researchers may understand the illness and its symptoms, it isn’t possible for them to understand the subtle complexities of daily living with the disease. The EPR’s own knowledge and his connection ^1^ with the wider MS community and their experiences provides an added depth to understanding the implications of the disease and how they may influence how the trial. This letter describes how the research team has used ‘the patient’ in the context of PPI (EPR and Patient Advisory Panel) to develop trial materials and procedures.

## Patient perspective

Every fortnight, our research team (principal investigator [PI], the post-doc and the research assistants) meet to discuss progress on the COB-MS Trial. This meeting keeps everyone updated and also allocates tasks for the upcoming weeks and months. My role at these meeting is to be the voice of the patient.

At these meetings there are many examples of how I share my lived experience of multiple sclerosis (MS). In this letter I chose two examples. The first highlighting the different knowledge areas of the team members, and the second demonstrating access into the patient community.

### Knowledge scope

At one of these meetings, the PI said they had prepared the Patient Participant Manual for the study and they had a sample of the booklet. It was beautiful. For me it looked like something I would be proud to have in my bookcase or even on the coffee table. The way the manual was manufactured was excellent. As a person with MS for 28 years, however, looking at this as a tool for making the intervention more effective for the participants, I knew it was only fit for the bin. Of course, I shared my opinion with the team - highlighting that it would not lie flat on the table, the pages were too slippery, and the gloss made it difficult to see what was written on the pages.

It surprised the team. At no stage did they realise the document they had produced would not be suitable for people like me, and why should they? No one else had my lived experience. Even though they knew all about MS, its symptoms and prognosis, they never lifted a book with MS hands.

Once they recovered from the surprise, we got down to the secondary benefit of having the patient at the table - I could share what I needed to make the manual more suitable. Some of the things I suggested were the importance of the type of paper, the binding, location of the page numbers, font and spacing – plus extras like a pouch for loose pages and extra forms (just in case). We then shared this format, along with the original, with the Patient Advisory Panel. They agreed with my opinion and added a few more suggestions.

What a success and a perfect example of why research should have a patient or potential recipient of a new therapy involved at all stages of the research. The researcher doesn’t know what they don’t know; and it is only through having the input of someone with the lived experience at the table they will know if they are going down the wrong path.

### Access to the community

At another meeting, we were discussing the recruitment of participants to the trial. This is always difficult, from what I am told, and when we had a small response to our initial press release, we began to talk about how else we can get in front of more people with MS.

Leaflets and posters in MS Society and GP offices was a start. Utilising my connection with a local radio station (Connemara FM), I did an interview about the study, my role in it and the type of people we needed. This brought in a few more people and revealed the power of the ‘patient story’. Subsequently, I used my connections within the MS community to get interviews with local and national press (e.g. Irish Independent, Irish Examiner and Farmers Journal, to name a few), as well as radio in areas we needed more participants.

The impact on recruitment was immediate. Within several weeks, we had enough to start the next stage of the trial and prepared to start the therapy. *Why was this successful?* I believe when you hear or read of someone who is going through a similar experience to your own, you form a bond, a camaraderie, which strengthens the trust you have in the message they share. I am one of them; and if I believe in it, then, perhaps, it will work for them, too.

### Embedded patient collaboration

Imagine an architect only asking the user of the building when the structure is nearly complete, where to locate the light switches, meanwhile none of the doors are wide enough for the user’s wheelchair. The recipient of the product, service, or therapy must be part of the process.

Developing anything new, which should have a long and useful life needs to be a collaborative venture – with all the stakeholders at the table, sharing equal status. We need more than engagement or participation, as these are additions to research, and not essential parts of the process. We need to be collaborators; patients don’t want to be there just to tick the box – *we* want to be there from the start, taking part in every decision.

The COB-MS program is about to be delivered to participants and the new manual will be used, which we hope will show people receiving the intervention that the research team understands what it is like to have MS and *our* particular needs.

I have shared two examples in this letter of how patient involvement has altered the course of this study. Some were small changes – and others bigger; but each one demonstrates to the participants in the trial that we understand what it is like to have MS. Every action is looked at through the lens of someone with the illness, who knows the role it plays in their daily experience. The challenge is how to measure the impact of each one of these on the trial.

In the last few months, this trial has also had to face the complication of the COVID-19 pandemic and its impact on research. I am an active part of the MS community here in Ireland, as well as in the UK and USA. I knew since its arrival the impact it would have on our study. Delivering the intervention in person would be an immense challenge. Online delivery would be the only route for most people with MS, because of the increased risk for this patient cohort.

Based on this knowledge, the team started to prepare for the possibility of changing the delivery method. We met with the PPI Advisory Panel to get their opinion and they agreed with the change. Moreover, the panel shared its experience of online therapies and groups, which helped us design the new format. This was combined with recommendations made in past, relevant research regarding how to use online platforms like that we are now using to deliver the program.

Once again, because a patient is ‘at the table’, it is possible to pre-empt potential challenges. *We* are also invested in the project, as we need it to be a success; and so, we can also provide concrete recommendations on how to proceed. We want and need to be collaborators. There for every decision, ensuring the focus remains on the patient first, with the goal of a better quality of life for those who receive the therapy.

### Challenges

In order to provide a balanced perspective of PPI in this project, it is useful to also mention any obstacles which had an impact on me and the research. As I am the first person to be employed in this type of role for the university, the induction process was not adequate. Even though there was an independent professional assessment of my needs, it did not translate into Human Resources putting in place the supports I needed to be effective in my role.

These included having a suitable office space, a location to store a mobility scooter and suitable chair, desk and computer. Some of these were resolved by the university and others were resolved by the pandemic and actions I had to take as an individual. Fortunately, I had the personal resources to resolve these, but this would not be the case for every patient and will act as an obstacle to effective PPI.

People who have chronic medical conditions, like multiple sclerosis, have financial support from the government. These payments link with access to medical support, such as the medical card in Ireland. If the role of EPR is paid, it could jeopardise these necessary supports, resulting in less involvement of patients due to this risk. This prevents a diverse range of people from being part of research, which will reduce the quality of the research.

Every team takes time to develop and form a cohesive whole. This team was no different. However, each team member wished to ensure the success of PPI in the trial. This desire to incorporate the patient in every aspect was led by the PI, ensuring the Patient Advisory Panel was used as often as required.

## Post-doctoral researcher experience

I’ve been conducting research with human participants for about 15 years now and one thing I can say about it is that recruitment is always a tricky process – especially when recruiting a very specific cohort, such as people living with MS. Consistent with previous efforts to recruit for other interventions, the research team and I proposed to advertise the trial through relevant newsletters and websites for people living with MS (e.g. *MS Ireland*), through occupational therapists working with us to deliver the intervention, as well as posters and information leaflets posted in relevant clinics. Being cognisant of the modern world, we also made a point to advertise through social media. We also developed a press release for our trial through our university’s PR department, in the hope that either local or national press might pick our study up. Though we garnered fair interest through referrals from specific societies and services, unfortunately, we didn’t gain much interest in light of our press release. It seemed something was missing. 

As I believe now, that ‘something missing’ was, in fact, a story to tell. Perhaps, from a researcher’s standpoint, the most important thing to convey about participating in an intervention, like COB-MS, is a general idea about who the program is for, what is involved, how it might help and how to make contact. Though this method is straight to the point, truth be told, it probably lacks that ‘human’ touch in which people can more easily ‘connect’ with the message – much like a story.

When our PPI member volunteered to take over the lead of advertising our study, the message ceased to be about the randomised nature of allocating participants, eligibility criteria or even about the outcome measures. All of that could be discussed later. For now, it was just about a single person with MS, telling their story – and when other people with MS heard that story (or even people who knew someone with MS – the message was shared), a connection was made and we started receiving emails and phone calls.

To be clear, our research team didn’t do anything wrong when we initially advertised the intervention. Indeed, the research team went about it much the same any other research team might. However, it’s not about what we did; rather, what we didn’t do sooner – and that’s giving the advertisement reins to our PPI from the very start. Sure, our PI or post-doc could have persevered to get on the radio or an interview for a paper; but that probably wouldn’t have done much to boost our sample either… because we don’t have MS. So, we don’t have that story to tell or the ability to connect with potential participants in that way; and that’s why PPI is so important to research programs like COB-MS.

### Challenges

Though I’ve always understood the potential benefits PPI could offer, I did have concerns upon hearing that PPI would be integrated within this research program; particularly, in light of conversations with researchers from other projects, as well as challenges PPI has presented me in some of my past research ventures. Primarily, I was concerned about how such involvement could impact the project’s timeline; and though that may be a reasonable concern in some contexts, what I strongly believe facilitated the successful implementation of PPI in our current research is the manner in which it was integrated. Our embedded patient researcher was involved in the research from the very beginning and was a part of a vast majority of the decision-making processes throughout the project’s life-cycle. Even when he wasn’t a part of it, he knew about it and the rationale behind it. If he had a problem with it, he would let us know quickly. Our embedded patient researcher works as part of the team, concurrently, which supports our timeline. To reiterate, I remain with concerns over having PPI integrated within research that I’m working on (given that the introduction of any additional consideration to a timeline could potentially lengthen it); but, that’s not as a result of PPI itself; rather, it’s down to the project’s design and how PPI is being implemented. If it is the goal of a researcher to integrate PPI within their project, I would highly recommend involving an ‘embedded patient researcher’ strategy, with someone who is there from start-to-finish and is genuinely involved, throughout, as a part of the research process. 

## Principal investigator experience

I believe that there are ethical reasons why patients should be included in the planning, design, and implementation of research. Within the scientific community, researchers tend to have strong opinions on the topic of PPI in research ( Boivin *et al.*, 2018). Though I have always valued PPI involvement in research, my “involvement” with PPI had been at somewhat of a distance before the COB-MS trial. In the context of this reflection, when referring to PPI activities I am including the work of our COB-MS PPI Advisory group and our Embedded Patient Researcher (RJ).

The effectiveness of PPI is strongest when people with lived experience of the condition being studied are involved as research partners ( Crocker *et al.*, 2018). When writing the funding application for the COB-MS feasibility trial, I wanted to be sure that there was a strong PPI through the trial. This was achieved through a variety of approaches but included a core PPI Advisory group, an Embedded Patient Researcher (person living with MS), and support throughout the process was received from PPI Ignite at NUI Galway. Though I anticipated that it might be a challenge to have a PPI member as a contract researcher, I believed that it would be key for this feasibility trial to have an experience-based expert contributing to decisions about the trial. As the PI I was also challenged because there was no precedent in the university for this type of contracted role. Though the university has training and supports available for research staff, the needs of someone taking on this position were likely to be different. Were I in a position to offer this type of role again I feel I would be much better prepared given the experience with this COB-MS trial.

 In the COB-MS trial we meet regularly, and all decisions are made together which ensures an openness and transparency within the research team. It can be a challenge when expectations, values and assumptions of researchers and PPI members do not match and this can impact on the experience of both parties ( O’Shea *et al.*, 2019) but this can be addressed through discussion and compromise.

PPI members as experts of their own condition, have experience that is invaluable in the design and running of clinical trials, as well as being aware of the needs, worries, and expectations of participants. As a PI, this is vital to the success of the trial. As a researcher and clinician, you can only understand so much and our PPI partners are really key to bridging that gap. Our PPI members are experience-based experts who contribute knowledge that is complementary to that of researchers on the trial ( Karazivan *et al.*, 2015) and have led to us making changes in a number of key areas. Two I will discuss here are 1) development of our participant handbook, and 2) participant recruitment. Having already read the reflections of the PPI contributor and the postdoc I will give my perspective (PI on the trial) of these examples.

### The participant handbook

The content of the handbook (which details the intervention and acts as a manual for participants and occupational therapists delivering the COB-MS) was developed prior to the beginning of the trial. Part of the process of developing the handbook involved consultation with people with MS and occupational therapists (see Hynes & Forwell, 2019). Feedback and focus groups with key stakeholders were key to the development process- in this case occupational therapists and people with MS. With feedback obtained the handbooks were updated and formatted- two versions of the handbook exist, one for people with MS and one for occupational therapists delivering the COB-MS. I was then happy to get them printed off and ready for participants.

A sample of the handbook was brought to the research team meeting for approval. Time and thought had been put into the design and layout so I had not anticipated any issues with it. The researchers were happy with the handbook, but it was our PPI contributor, the embedded patient researcher (RJ), who immediately spotted a number of important issues that needed to be addressed before we printed the batch of 100+ handbooks for our participants. 

Given the changes suggested, we decided to have a meeting with our wider PPI advisory group where we brought different handbook samples and discussed areas that needed to be changed or included in the final version. Attending that meeting very much solidified the importance of PPI in this feasibility trial for me. The changes that were made ensured that the intervention was more accessible for participants and showed that the research team had an awareness of what would help people participate fully. These issues would not have been spotted were it not for the PPI members on the trial. It can be difficult when you receive negative feedback on something you have designed and formatted but if the end result means that the handbook facilitates participation, and does not become another barrier then there is no doubt that it is worth it! Fostering a culture of openness and kindness within the group allows for these difficult discussions to take place easily. The handbook is now being successfully used by participants in the trial and informal feedback has been very positive.  

### Participant recruitment

As we were recruiting participants, there were some geographical areas that we were finding it difficult to recruit from. We did a call through the MS Society and through the occupational therapists working in the area but were unable to recruit adequate numbers in some areas. Our embedded patient researcher decided to get involved in the recruitment process and took it upon himself to do interviews for radio and print media, with a specific focus on the difficult to recruit areas. Our wider PPI Advisory Panel also helped in suggestion where and how we might best target our recruitment in those areas. Having someone living with the condition talk about the COB-MS trial allowed people listening to hear the experience of someone living with the same condition and explain the research in a way that is meaningful and understandable to them. This effect on recruitment has been reported elsewhere with PPI interventions having a modest but significant increase the odds of participant enrolment ( Crocker *et al.*, 2018; Domecq *et al.*, 2014). MS Ireland were key to successful recruitment and their networks allowed us to reach a wide group of people. The addition of recruiting through print and radio with EPR meant that we could reach people with MS who were not linked with MS Ireland, which was an important part of our planned recruitment strategy. 

There are still barriers that exist to recruiting under-served communities in research and clinical research has for the most part failed to adequately address this issue. Involving PPI members from these under-served communities could be a way of trying to increase the diversity of our participants ( Smits *et al.*, 2020). The INCLUDE roadmap, developed by the NIHR ( NIHR, 2020) provides an overview of the stages of the research process where researchers can act to include more diverse groups of people, particularly those defined as “under-served”. This is a goal that we have for future PPI work in MS.  There is a significant number of people living with various health conditions, including MS, that choose not to be a part of the associated organisation. It is important to develop ways of reaching these groups and allowing the opportunity to participate in research. We, in the COB-MS trial, acknowledge that this is an area that we need to do better in, but we are working towards this, and have incorporated this into our dissemination plan. 

## Conclusion

I have given two examples here of PPI in the COB-MS trial. There are many more we could have discussed in this Open Letter. As a research team we plan to capture as much of the PPI activity as possible in the COB-MS trial through the completion of a PPI framework/checklist that we have adapted for the trial. We will report on this along with the other dissemination activities. There are those who feel that in order to be taken seriously, we must evaluate involvement as we do other research processes, though this view is being debated more recently. If PPI is viewed as a part of the research process then maybe there is not the same need to measure its impact and we would be better served capturing the negative and positive aspects of the process instead ( Russell *et al.*, 2020). 

One overarching reflection is that by having a strong PPI input in the COB-MS trial we have never forgotten why we are doing the research we are doing, and how it might impact the lives of those we are serving through our research. 

## Discussion

In this letter we have examined the positive and negative aspects of incorporating the patient in a trial. To effectively share this experience we have looked at the impact on the patient, a post-doc researcher and the Principal Investigator on the trial. Due to their different viewpoints each person highlighted a different aspect of PPI in the two areas we have reviewed.

Over the course of the first twelve months of this trial the EPR has attended every meeting of the Trial Management Committee, resulting in his being part of every decision taken. Patients have unique needs which can be complex when including as collaborative partners in research. These challenges have been discussed in this letter, but were overcome through the cooperation of the whole team. In effect, a bonding exercise, which has led to small and large changes to how the trial proceeded. In this letter we have highlighted two of these changes, the Participant Manual and Recruitment.

The Participant Manual had been developed for the initial trial and was felt to still be suitable for this Feasibility Study. The EPR was able to highlight weaknesses in the format of the document which made it unsuitable for people living with MS. By using his lived experience and the knowledge of the Patient Advisory Panel substantial changes were made to the Participant Manual, ensuring the participants in the trial had learning materials suitable for people living with MS.

Recruitment is always a challenge. Using the traditional methods of recruitment, it slowed down once we had 50% of participants recruited. When the EPR took on the role of recruiter by sharing his experience on radio, newspapers and online, it was possible to recruit the remaining 50% of participants. This sharing of a personal experience of MS ensured the trial had a more diverse group of participants (geographic, age and socio-economic) than the traditional approach.

## Reflection

Introducing patient involvement in research is gathering pace internationally. This trial has significant input of patients at all stages. The writers of this letter acknowledge integrating PPI can have bureaucratic obstacles, but by sharing these challenges, how they have been overcome, and the positive impact on the trial of integrating the patient in all stages, we hope more research will focus on collaboration with the ultimate beneficiaries of the trial.

## Data availability

No data are associated with this article.
