# Supplementary material for: Twelve months into a feasibility trial: reflections on three experiences of public and patient involvement in research
Source: HRB Open Res. 2021 Jul 22;4:11. Originally published 2021 Jan 28. [Version 2] doi: 10.12688/hrbopenres.13205.2 (PMC8320708; doi:10.12688/hrbopenres.13205.2)
Supplement: Peer review history

# Peer review: Reviewer response for version 2

**DOI:** 10.21956/hrbopenres.14557.r29892

**Published:** 2021-08-20

**Authors:** Ingrid Klingmann

**Affiliations:** 1 European Forum for Good Clinical Practice (EFGCP), Brussels, Belgium

Altogether, this is now a very interesting, informative and encouraging Open Letter. 

 Just a few details to make it perfect: 

**Abstract:**

 … *the impacts that have been resulted from of PPI input into the trial.* Please check, I think some words have not been deleted. 

**Introduction, last paragraph:**

 ... *an added depth to understanding the implications of the disease and how they may influence **how** the trial. *Delete "how".

 A big Thank You to the authors for this excellent contribution to promoting PPI.

Does the article adequately reference differing views and opinions?

Yes

Are all factual statements correct, and are statements and arguments made adequately supported by citations?

Yes

Is the Open Letter written in accessible language?

Partly

Where applicable, are recommendations and next steps explained clearly for others to follow?

Partly

Is the rationale for the Open Letter provided in sufficient detail?

Yes

Reviewer Expertise:

Clinical research, medicines development sciences, ethics, patient engagement.

I confirm that I have read this submission and believe that I have an appropriate level of expertise to confirm that it is of an acceptable scientific standard.

# Peer review: Reviewer response for version 2

**DOI:** 10.21956/hrbopenres.14557.r29893

**Published:** 2021-08-04

**Authors:** Marie-Luise Dierks, Jonas Lander

**Affiliations:** 1 Institute for Epidemiology, Social Medicine, and Health Systems Research, Hannover Medical School, Hannover, Germany; 2 Institute for Epidemiology, Social Medicine and Health Systems Research, Hannover Medical School, Hannover, Germany

Thank your for the opportunity to re-review this, it seems that the authors have carefully considered the previous suggestions, so we are happy to approve this version without further reservations.

Does the article adequately reference differing views and opinions?

Yes

Are all factual statements correct, and are statements and arguments made adequately supported by citations?

Yes

Is the Open Letter written in accessible language?

Yes

Where applicable, are recommendations and next steps explained clearly for others to follow?

Partly

Is the rationale for the Open Letter provided in sufficient detail?

Partly

Reviewer Expertise:

Patient Public Involvement in medical research, I’m not an expert for MS.

We confirm that we have read this submission and believe that we have an appropriate level of expertise to confirm that it is of an acceptable scientific standard.

# Peer review: Reviewer response for version 2

**DOI:** 10.21956/hrbopenres.14557.r29891

**Published:** 2021-07-28

**Authors:** David Evans

**Affiliations:** 1 Department of Health and Social Sciences, University of the West of England, Bristol, UK

The authors have very fully taken on board the question I set them in my first review regarding whether there were not challenges they might learn from as well as the benefits of PPI. They have added reflections and learning from challenges from the three authors' different perspectives, which has in my view marked improved the letter. I have no further comments to make on what is now a very useful reflective letter which other research teams can learn from.

Does the article adequately reference differing views and opinions?

Partly

Are all factual statements correct, and are statements and arguments made adequately supported by citations?

Yes

Is the Open Letter written in accessible language?

Yes

Where applicable, are recommendations and next steps explained clearly for others to follow?

Yes

Is the rationale for the Open Letter provided in sufficient detail?

Yes

Reviewer Expertise:

Patient and public involvement in health services research.

I confirm that I have read this submission and believe that I have an appropriate level of expertise to confirm that it is of an acceptable scientific standard.

# Peer review: Reviewer response for version 1

**DOI:** 10.21956/hrbopenres.14354.r29061

**Published:** 2021-04-09

**Authors:** Ingrid Klingmann

**Affiliations:** 1 European Forum for Good Clinical Practice (EFGCP), Brussels, Belgium

This open letter aims at presenting a practical example for successful engagement of a patient living with the disease into the academic research team from the perspective of the patient, the PhD researcher and the principal investigator. The structure of the open letter enables the presentation of these different experiences. The format of an open letter is different from that of a typical research publication with introduction, material and methods, results, discussion and conclusions. But also this open letter would benefit from a discussion section that would reflect the presented experiences individually and in their entirety. The description of the "successes" are not really aligned: the PhD researcher seems to have no appreciation of the patient's contribution to the improvement of the handbook; the PI does not comment on why the patient's contribution to the handbook enabled considerable improvements to a version that originally had already patient input. And there is no discussion of the fact that all three authors present different reasons why the patient's involvement in recruitment efforts was successful. And there is no critical discussion about what could have been done better in that collaboration or a recommendation to the reader on what to avoid. Ideally, this could have resulted in joint conclusions from the three authors. 

 There are some few spelling errors, an important one is the varying spelling of the PI which should read "principal investigator".

 And the open letter would benefit from a few more citations in the introduction or the discussion on the current state of knowledge and thinking about patients with various levels of knowledge about the two aspects: living with the disease and clinical research methodology and the types of tasks they can most efficiently contribute to.

Does the article adequately reference differing views and opinions?

Yes

Are all factual statements correct, and are statements and arguments made adequately supported by citations?

Yes

Is the Open Letter written in accessible language?

Partly

Where applicable, are recommendations and next steps explained clearly for others to follow?

Partly

Is the rationale for the Open Letter provided in sufficient detail?

Yes

Reviewer Expertise:

Clinical research, medicines development sciences, ethics, patient engagement.

I confirm that I have read this submission and believe that I have an appropriate level of expertise to confirm that it is of an acceptable scientific standard, however I have significant reservations, as outlined above.

# Response

**Published:** 2021-07-14

**Authors:** Sinéad Hynes

**Affiliations:** NUI Galway, Ireland

**Many thanks for your review and comments on our open letter. We considered each, in turn, and have amended the manuscript in light of these considerations. Other, specific comments and questions we answer here.**

 Comment 1: This open letter would benefit from a discussion section that would reflect the presented experiences individually and in their entirety. The description of the "successes" are not really aligned: the PhD researcher seems to have no appreciation of the patient's contribution to the improvement of the handbook; the PI does not comment on why the patient's contribution to the handbook enabled considerable improvements to a version that originally had already patient input. And there is no discussion of the fact that all three authors present different reasons why the patient's involvement in recruitment efforts was successful.

**Response 1: A discussion section has been added to more comprehensively reflect the experiences of the individual team members.**

 Comment 2: There is no critical discussion about what could have been done better in that collaboration or a recommendation to the reader on what to avoid. Ideally, this could have resulted in joint conclusions from the three authors. 

**Response 2:** **Many thanks for this comment, consistent with Reviewers 1 and 2’s comments, we have included discussion of challenges and have made important additions to the discussion to clarify the team members’ perspectives.**

 Comment 3: There are some few spelling errors, an important one is the varying spelling of the PI which should read "principal investigator".

**Response 3:** **All spelling and typographical errors have been amended.**

 Comment 4: And the open letter would benefit from a few more citations in the introduction or the discussion on the current state of knowledge and thinking about patients with various levels of knowledge about the two aspects: living with the disease and clinical research methodology and the types of tasks they can most efficiently contribute to.

**Response 4: A variety of relevant citations are included, where appropriate, including: Boivin *et al.* (2018), Crocker *et al.* (2018), Domecq *et al.* (2014), O’Shea *et al.* (2019), Russell *et al.* (2020) and Smits *et al.* (2020). In keeping with the focus of the manuscript we have kept a reflective and accessible approach to the manuscript which is written by PPI member. For this reason, we chose to only include the most relevant citations.**

# Peer review: Reviewer response for version 1

**DOI:** 10.21956/hrbopenres.14354.r29060

**Published:** 2021-03-23

**Authors:** Marie-Luise Dierks, Jonas Lander

**Affiliations:** 1 Institute for Epidemiology, Social Medicine, and Health Systems Research, Hannover Medical School, Hannover, Germany; 2 Institute for Epidemiology, Social Medicine and Health Systems Research, Hannover Medical School, Hannover, Germany

This open letter is a valuable and novel way of sharing experiences about PPI so we guess its indexing will be an important contribution to the field.

 We have a few bullet point comments for the beginning of the letter:

- It would be good to be clearer in the Intro what you mean by “PPI member”, only patient/public or all involved in the process?
- Why are personalised experiences important in research about PPI, what do they contribute to the debate?
- What are “production values”?
- Can you provide a short explanation about a logical structure for this letter, i.e. who discusses which aspect and how do they complement each other?

The writing and ideas in each of the 3 perspectives seem fine and logical, we don’t have much to add here.

 The conclusion, however, could be slightly improved, you do mention the aspect of PPI evaluation, but we don’t see an overall conclusion from the 3 perspective, e.g. the most positive but also negative aspects of your PPI, i.e. were there things that were challenging, that caused disagreement, workload, etc.? Also, can you think again about your overall message to those planning PPI, what do you want them to do (Not sure if this has already been made clear so far).

Does the article adequately reference differing views and opinions?

Yes

Are all factual statements correct, and are statements and arguments made adequately supported by citations?

Yes

Is the Open Letter written in accessible language?

Yes

Where applicable, are recommendations and next steps explained clearly for others to follow?

Partly

Is the rationale for the Open Letter provided in sufficient detail?

Partly

Reviewer Expertise:

Patient Public Involvement in medical research, I’m not an expert for MS.

We confirm that we have read this submission and believe that we have an appropriate level of expertise to confirm that it is of an acceptable scientific standard, however we have significant reservations, as outlined above.

# Response

**Published:** 2021-07-14

**Authors:** Sinéad Hynes

**Affiliations:** NUI Galway, Ireland

**Many thanks for your review and comments on our open letter. We considered each, in turn, and have amended the manuscript in light of these considerations. It is our hope this letter will help both researchers and patients to see the value of including the patient integration throughout the research programme’s lifespan. With this in mind – as well as your valuable comments, we have added more content to the letter to highlight what we mean by PPI in the trial. Patients and their representative organisation have been involved in every aspect of the study, including this letter. We have amended the letter to include some of the challenges faced by researchers and patients to provide more balance. Your comment about having conclusions from each perspective has also made the document richer; highlighting the different viewpoints. Other, specific comments and questions we answer here.**

 Comment 1: It would be good to be clearer in the Intro what you mean by “PPI member”, only patient/public of all involved in the process?

**Response 1: On Page 2 we have included the following text: “The patient is a common thread throughout the trial. Upon grant application, the MS Society of Ireland and the PPI Ignite project were advisors on how best to incorporate PPI. This led to the employment of an Assistant Researcher living with MS (from here on described as an Embedded Patient Researcher [EPR]), a Patient Advisory Panel and patients being members of the Trial Steering Committee and Trial Management Committee.”**

 Comment 2: Why are personalised experiences important in research about PPI, what do they contribute to the debate?

**Response 2: Introduction and the reflection has now addressed this query, thank you. We have included at the end of the document a Reflection section which clarifies the importance of personal experiences in the context of PPI: “Introducing patient involvement in research is gathering pace internationally. This trial has significant input of patients at all stages. The writers of this letter acknowledge integrating PPI can have bureaucratic obstacles, but by sharing these challenges, how they have been overcome, and the positive impact on the trial of integrating the patient in all stages, we hope more research will focus on collaboration with the ultimate beneficiaries of the trial.”**

 Comment 3: What are “production values”?

**Response 3: Production values referred to the quality of the patient handbook developed for the trial. This line has been reworded to enhance clarity.**

 Comment 4: Can you provide a short explanation about a logical structure for this letter, i.e. who discusses which aspect and how do they complement each other?

**Response 4: Trial teams are comprised of people coming from different backgrounds, which leads to unique perspectives on events. The writers of this Open Letter felt it would be constructive to share the actual experience of the patient, the post-doc researcher and the PI, individually. By sharing these unique experiences it should help the wider discussion of PPI and how it impacts research behaviour. As a patient, it is helpful to know why the other team members contribute to the team discussions in a particular manner. Equally, the researchers need to understand the impact of this experience on an Embedded Patient Researcher.**

 Comment 5: The conclusion, however, could be slightly improved, you do mention the aspect of PPI evaluation, but we don’t see an overall conclusion from the 3 perspective, e.g. the most positive but also negative aspects of your PPI, i.e. were there things that were challenging, that caused disagreement, workload, etc.? Also, can you think again about your overall message to those planning PPI, what do you want them to do (Not sure if this has already been made clear so far).

**Response 5:** **Many thanks for this comment, consistent with Reviewer 1’s comment, we have included discussion of challenges and have made important additions to the concluding section to clarify individual perspectives and our cumulative message.**

# Peer review: Reviewer response for version 1

**DOI:** 10.21956/hrbopenres.14354.r28911

**Published:** 2021-03-04

**Authors:** David Evans

**Affiliations:** 1 Department of Health and Social Sciences, University of the West of England, Bristol, UK

This is an interesting and informative open letter, detailing the experience of patient and public involvement (PPI) in a feasibility trial from the perspective of a patient employed as a contract researcher, a post-doc researcher and the principal investigator. The letter is valuable in providing convincing reflections of the benefits to the research of the patient member's involvement. My only question is whether the authors have avoided reflection on any difficulties or challenges they may have faced in employing a patient as a contract researcher. Having done so myself on several projects, I have experienced both similar benefits to those described here, but inevitably I have also experienced challenges. They have ranged from bureaucratic obstacles in terms of organisational HR, finance and research governance policies, through to team dynamics. I would be surprised if the authors experienced no challenges whatsoever in these regards, and if they did, it would be helpful if they reflected on these as well as the clear benefits of this PPI. If they really experienced no challenges, then it would be good just to document this.

Does the article adequately reference differing views and opinions?

Partly

Are all factual statements correct, and are statements and arguments made adequately supported by citations?

Yes

Is the Open Letter written in accessible language?

Yes

Where applicable, are recommendations and next steps explained clearly for others to follow?

Yes

Is the rationale for the Open Letter provided in sufficient detail?

Yes

Reviewer Expertise:

Patient and public involvement in health services research.

I confirm that I have read this submission and believe that I have an appropriate level of expertise to confirm that it is of an acceptable scientific standard, however I have significant reservations, as outlined above.

# Response

**Published:** 2021-07-14

**Authors:** Sinéad Hynes

**Affiliations:** NUI Galway, Ireland

Many thanks for your review and comments on our open letter. We appreciate the feedback. To address the question from your review, the open letter has now been amended to include challenges faced on pages 4 -7.
